# The microbiota of wooden cheese-ripening boards is a rich source of antimicrobial-producing bacteria against *Listeria monocytogenes*

**DOI:** 10.1128/spectrum.02936-25

**Published:** 2026-01-07

**Authors:** Yuxing Chen, Ibrahim Zuniga Chaves, Jennifer C. McClure, Garret Suen, TuAnh N. Huynh

**Affiliations:** 1 Food Science Department, University of Wisconsin–Madison 5229 , Madison, Wisconsin, USA; 2 Department of Bacteriology, University of Wisconsin–Madison 5229 , Madison, Wisconsin, USA; 3 USDA-ARS Dairy Forage Research Center 57844 , Madison, Wisconsin, USA; University of Minnesota Twin Cities, St. Paul, Minnesota, USA

**Keywords:** antimicrobial peptides, wooden cheese boards, *Listeria monocytogenes*

## Abstract

Wooden boards are essential tools in cheese ripening, and there is accumulating evidence that they have antimicrobial effects against foodborne bacterial pathogens, such as *Listeria monocytogenes*. However, poor bacterial recovery of bacteria from wood can confound the quantification of pathogen burden. To assess *L. monocytogenes* survival on wooden cheese boards, we applied a disruptive grinding method and tracked native board-associated bacterial counts as controls. Our data revealed that *L. monocytogenes* declines on clean zones of wooden boards but can replicate on areas where there is suitable cheese. Our microbiota analysis revealed diverse bacterial communities on wooden board surfaces, with a prominent presence of bacteria in the genera *Brevibacterium*, *Brachybacterium*, and *Staphylococcus*. We further identified seven bacterial species that inhibit *L. monocytogenes*, belonging to *Bacillus*, *Staphylococcus*, and *Serratia* phyla, in addition to *Lactococcus lactis*. One isolate, *Bacillus safensis*, was found to be a potential biocontrol agent, potently inhibiting *L. monocytogenes* via secreted antimicrobial factors. Our genomic, bioinformatic, and biochemical analyses indicate that those factors are likely antimicrobial peptides encoded by multiple biosynthetic gene clusters, many of which have not been characterized. A sub-inhibitory concentration of *B. safensis* supernatant induces a significant downregulation of prophage elements and an upregulation of antimicrobial stress responses in *L. monocytogenes*. Taken together, our findings indicate that the wooden board microbiota is a rich source of antimicrobial-producing bacteria with potential applications in foodborne pathogen control strategies.

**IMPORTANCE:**

Despite stringent food safety measures, *L. monocytogenes* foodborne outbreaks remain frequent with high hospitalization and mortality rates. Removal of *L. monocytogenes* from food processing environments is extremely challenging because this pathogen is ubiquitous and encodes a wide array of stress response mechanisms that enable it to thrive under harsh conditions. Our study found that clean wooden boards used in cheese ripening inhibit *L. monocytogenes*, causing a noticeable decline in pathogen population following surface inoculation. Bacterial communities on wooden cheese boards are rich and diverse and harbor many species that produce antimicrobial compounds against *L. monocytogenes*, with the example of a new *B. safensis* isolate. Therefore, the wooden cheese-ripening board microbiota is a promising source for future antimicrobial discovery efforts.

## INTRODUCTION

Wooden boards have long been used for cheese ripening and remain popular among artisanal cheese makers, accounting for the ripening of ~500,000 tons of cheese in Europe every year (1). The porous and hygroscopic properties of wood are considered essential in the development of cheese sensory quality during ripening. Indeed, cheese made with wooden tools has been reported to exhibit a richer volatile compound profile than cheese made with other tools, due to the impact of wood on cheese properties (2). Wood used in cheese ripening is typically dried to 15%–18% humidity without chemical treatments. Wood with high humidity might support mold or *Pseudomonas fluorescens*. By contrast, dry wood might promote the development of *Serratia* species, which cause thick, strong rinds and red defects (1). Therefore, cheese makers often intentionally select woods with suitable hydrometry to achieve desirable levels of cheese humidity and ripening kinetics.

Wooden boards harbor a surface microbiota that is rich in composition and diverse among boards (1, 3–6). For instance, a comprehensive survey of wooden boards in 18 dairy facilities in Sicily, Italy, revealed at least 43 bacterial genera on each board (5). Although those boards exhibited distinct microbiota compositions, some bacterial groups are commonly present in significant abundances, including *Staphylococcus*, *Brevibacterium*, and *Corynebacterium*. Interestingly, these three genera were also found to be dominant in a survey of wooden cheese boards in Wisconsin, United States (4). Given that these bacteria are among the core members of the cheese rind microbial community (7), it remains unclear what microorganisms are native to wood that transfer to the cheese surface.

Bacteria on wooden board surfaces are considered a major source of cheese rind microbiota whose metabolic activities drive cheese flavor development (1, 8). The biofilm formed by these bacteria is stable and can withstand some sanitation treatments. This persistence has raised food safety concerns, highlighted by a brief US Food and Drug Administration ban of wooden boards in cheese ripening in the United States. Although this ban was quickly reversed, and wooden boards have a long history of safe use, dairy processing facilities are frequently contaminated with foodborne pathogens that may transmit to food contact surfaces. Therefore, thorough assessments of pathogen behavior on cheese boards are necessary to guide ripening process design.

*Listeria monocytogenes* is an invasive foodborne pathogen that is frequently associated with dairy product outbreaks. *L. monocytogenes* isolates from dairy products are particularly well-adapted to host conditions and gut colonization. A majority of those isolates are of clonal complex 1, which is hyper-virulent and strongly associated with human clinical samples (9). *L. monocytogenes* is ubiquitously present in dairy farms and is prevalently shed by cattle and therefore easily contaminates raw milk and dairy processing plants (10–13). Although pasteurization is effective at killing *L. monocytogenes*, this pathogen can contaminate dairy products at any processing step following pasteurization (14). The persistence of *L. monocytogenes* in dairy processing plants is well documented, and this pathogen can be detected at many locations within a plant, especially in difficult-to-reach areas such as drains, cracked surfaces, joints of equipment, and conveyor belts (14). From a physiological standpoint, *L. monocytogenes* has an array of stress response mechanisms that enable its persistence under dairy processing conditions and replication in dairy products. For instance, the general stress response regulator, σ^B^, is activated by many stress conditions and upregulates more than 200 genes to adapt to low pH, high osmolarity, and refrigeration, all of which are relevant in dairy production (15). Soft cheeses are particularly permissive to *L. monocytogenes* replication due to comparatively high humidity and neutral pH. Furthermore, *L. monocytogenes* is notorious for robust biofilm formation on different materials, such as glass, stainless steel, polystyrene, and polytetrafluoroethylene. These biofilms provide additional protection against antimicrobial treatments (16, 17).

Despite the widespread presence of *L. monocytogenes* in dairy processing plants, wooden boards have not been identified as the source of *L. monocytogenes* contamination in cheese. On the contrary, there is evidence for the antimicrobial effect of wooden boards. For instance, wood transfers significantly less *L. monocytogenes* to cheese than plastic and glass (18). Furthermore, *L. monocytogenes* burdens have been shown to significantly reduce following inoculation on native wooden boards (19). Interestingly, heat-inactivated wooden boards allow for *L. monocytogenes* replication, suggesting that the board surface microbiota confers inhibition (19). However, since *L. monocytogenes* recovery rate from wood is poor, at no more than 30% even with destructive methods (20), alternative experimental designs are necessary to accurately quantify *L. monocytogenes* survival rates on wooden boards. Nevertheless, a recent study found that culturable bacteria from wooden boards inhibit *L. monocytogenes* in laboratory culture, although antagonistic bacteria have not been systematically identified (21).

In this study, we aimed to assess *L. monocytogenes* survival on the wooden cheese board surface and to systematically identify board-associated bacteria that inhibit *L. monocytogenes*. To address the technical challenge of poor bacterial recovery from wood, we concurrently tracked both *L. monocytogenes* and wooden board-associated bacteria as controls. We found *L. monocytogenes* burdens to decline in most instances on sanitized and native boards, but at different rates and extents among boards. In one exception, we observed an increase in *L. monocytogenes* abundances relative to board-associated bacteria, although cheese residues likely contributed to this increase. From a small set of wooden boards from three cheese makers, we found total microbial DNA from wood shavings to be highly abundant in genera belonging to *Staphylococcus*, *Brevibacterium*, and *Brachybacterium*, consistent with two previous surveys of wooden boards elsewhere and with cheese rind microbiota (4, 5). However, our taxonomic survey of live, culturable bacterial communities from those boards revealed additional genera that are not typically associated with cheese rind, such as *Bacillus*. Exploiting this microbial diversity, we identified six bacterial species of the *Bacillus*, *Staphylococcus*, *Lactococcus*, and *Serratia* genera that inhibit *L. monocytogenes*. We focused on a novel isolate of *Bacillus safensis* as a potential biocontrol candidate and found it to potently inhibit *L. monocytogenes*, likely through secreted antimicrobial peptides. In response, *L. monocytogenes* significantly downregulated the prophage and monocin elements. Taken together, our findings indicate that the wooden cheese ripening board microbiota is a rich source of natural antimicrobials against *L. monocytogenes*.

## RESULTS

### Total microbial DNA from wooden boards harbors a diverse microbiota with similar compositions to cheese rind

**Fig 1:**
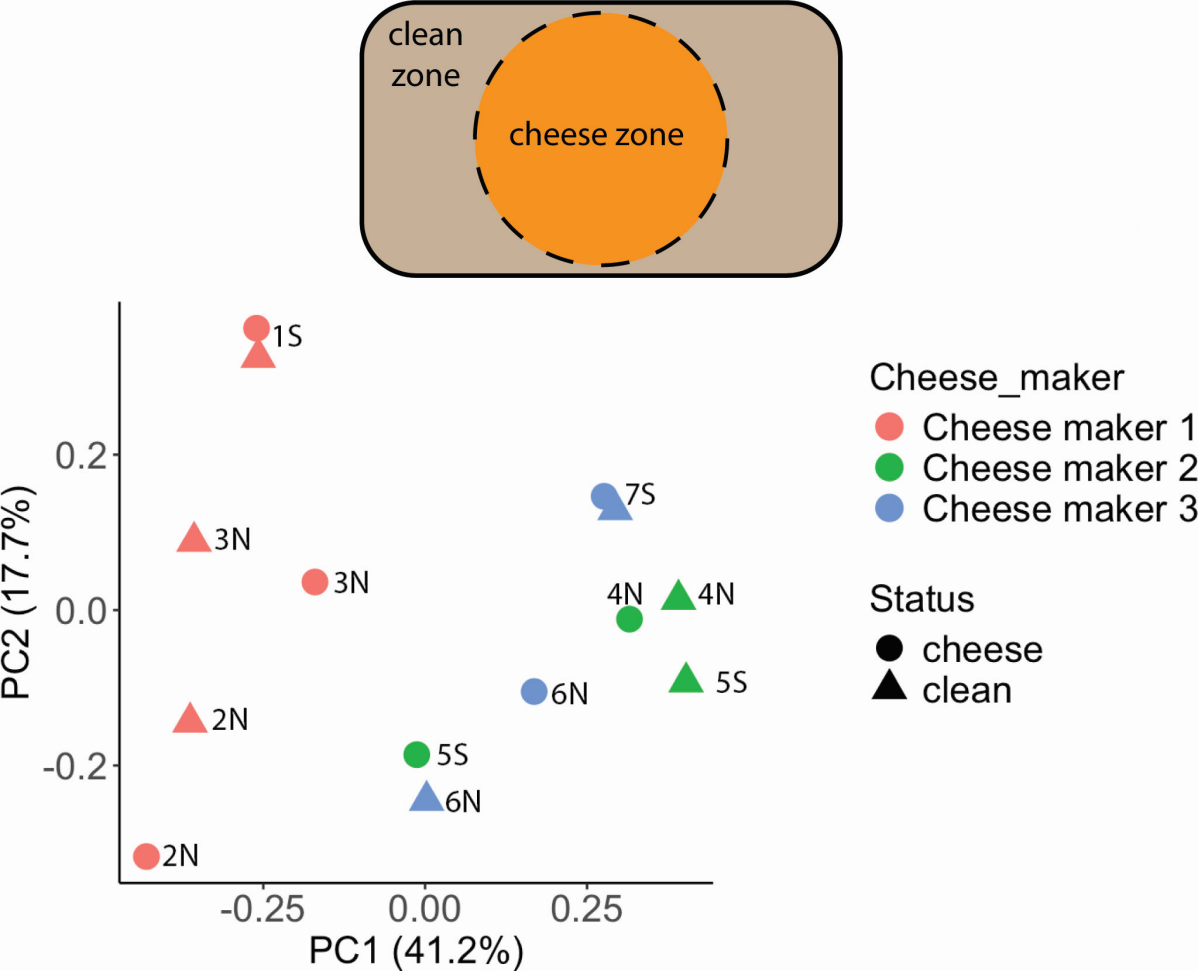
Wooden boards harbor distinct microbiota compositions. Top panel: clean zone and cheese zone on each wooden board. Bottom panel: principal component analysis of bacterial communities on wooden boards from three cheese makers. Each data point represents the microbiota composition of a native or sanitized wooden board from a cheese maker. Each board is marked with the board number, N indicates native boards, and S indicates sanitized boards.

Despite research interests in the microbial ecology of wooden cheese boards, only two studies have systematically determined the wooden board microbiota by next-generation sequencing (4, 5). To expand our knowledge of the microbial diversity on wooden cheese boards, we obtained seven wooden boards from three different cheese makers in Wisconsin, United States. These cheese makers are distinct from those surveyed in a previous study (4). Wooden boards are typically sanitized after cheese ripening, and we predicted that sanitation would significantly impact board microbiota. Therefore, we also obtained sanitized boards from each cheese maker. Boards from cheese maker 1 were made of cedar, including board 1 (sanitized), board 2 (native, used for a smear-ripened cheese), and board 3 (native, used for a hard cheese). Boards from cheese maker 2 were made of pine, including board 4 (native, used for a hard cheese) and board 5 (sanitized). Boards from cheese maker 3 were also made of pine, including board 6 (native, used for a hard cheese) and board 7 (sanitized). We noticed that each board had a circular cheese mark where a cheese block had been placed during ripening, as mapped out in Fig. 1. We therefore analyzed the bacterial compositions of “clean” zones separately from “cheese” zones on each board to avoid the confounding presence of cheese microbiota in our analyses.

We first performed a taxonomic survey using board wood shavings as previously described (4). Briefly, total DNA was extracted from the top 2 mm of each board, obtained by grinding, and subjected to short-read Illumina-based next-generation sequencing of the 16S rRNA gene. After denoising and decontamination, we obtained a total of 183,918 high-quality reads and grouped them into 62 operational taxonomy units (OTUs). These OTUs belong to 12 phyla, 16 classes, 44 orders, 72 families, and 93 genera. Due to the limited availability of boards, we did not obtain sufficient biological replicates of each board type to reliably compute α-diversity indices. However, we found these communities to have both rich and diverse microbiota, with each board harboring between 8 and 58 genera (median of 22).

**Fig 2:**
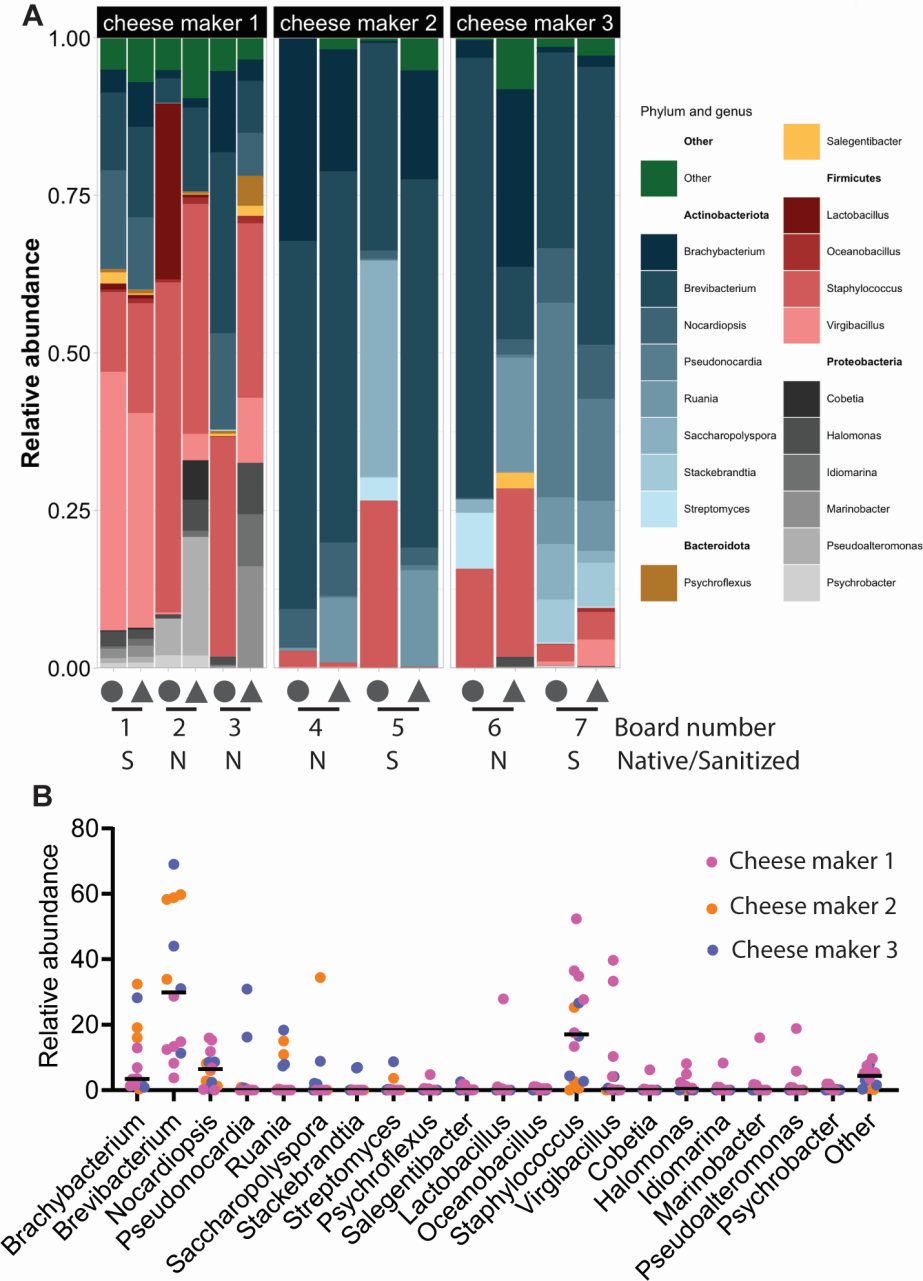
Bacterial communities on wooden boards are diverse. Relative abundances of the twenty most abundant phyla on seven wooden boards are organized for each board (**A**) or each phylum (**B**). In panel A, the circle symbol indicates the cheese zone, and the triangle indicates the clean zone on each board. Bars in panel B are median abundances.

Overall, the bacterial compositions from board wood shavings were reminiscent of cheese rind microbiotas and consistent with previous surveys of wooden board microbiotas (4, 5, 7). The three most abundant bacteria across all boards belonged to three genera: *Staphylococcus* (from 0.06% to 52%, median 17%), *Brevibacterium* (from 4% to 69%, median 30%), and *Brachybacterium* (from 0.4% to 32%, median 3.5%) (Fig. 2). As expected, bacterial compositions were distinguishable for wooden boards from different cheese makers, although those from cheese maker 1 were the most dissimilar (Fig. 1). From each cheese maker, microbiota composition was also distinguishable between native and sanitized boards, with most notable changes in the abundances of *Staphylococcus* and *Virgibacillus*.

While sharing some common bacteria, each board in our microbiota survey exhibited a unique composition (Fig. 2). Two native boards from cheese maker 1 were dominated by *Staphylococcus* (13% and 52%), *Brachybacterium* (1% and 13%), and *Brevibacterium* (4% and 29%). We also noticed that cheese blocks from native board 2 had a high abundance of *Lactobacillus* (~28%), likely transferred from cheese. Somewhat distinct from others, board 3 was also abundant in *Nocardiopsis* (7%–10%). By comparison, the sanitized board from this cheese maker (board 1) was significantly reduced in *Staphylococcus* and substantially increased in *Nocardiopsis*.

Boards from cheese maker 2 (boards 4 and 5) were dominated by *Brevibacterium* (34%–60%) and *Brachybacterium* (16%–32%), with an additional abundance of *Nocardiopsis* (1.2%–8%). Additionally, *Ruania* was abundant on clean blocks from this cheese maker (11%–15%), but not on cheese blocks, suggesting that these bacteria are of wood or environmental origins.

Wooden boards from cheese maker 3 (boards 6 and 7) were overall abundant with *Brachybacterium* (1%–28%), *Brevibacterium* (11%–69%), and *Staphylococcus* (3%–27%). As observed in cheese maker 1, *Staphylococcus* was much reduced in abundance on a sanitized board than on a native board (~3% compared with ~20%, respectively). By contrast, *Nocardia* and *Pseudonocardia* were much more abundant on a sanitized board from this cheese maker (~8% and ~23%, respectively).

### The compositions of live bacteria on wooden boards are distinct from total DNA footprints

Our taxonomic survey of wood grindings revealed a high prevalence of *Staphylococcus*, *Brevibacterium*, and *Brachybacterium*. These bacteria are easily grown under laboratory conditions and have distinct colony morphologies (22). However, when we isolated and identified bacteria from cheese boards using 16S rRNA sequencing, we rarely found *Brevibacterium*, although we still recovered *Brachybacterium* and *Staphylococcus*. Similarly, we frequently isolated *Bacillus* and *Lactococcus*, despite their extremely low abundances in our microbiota analysis. This led us to consider whether the total DNA extracted from wood grindings accurately reflects the live bacterial communities present on board surfaces after cheese ripening.

As a preliminary assessment of live bacteria on wooden boards, we used agar media to recover bacteria from the same wood shaving suspensions used in our initial survey. Based on a previous study indicating that most cheese rind bacteria are culturable (7), we plated these suspensions on TSA (rich, non-selective) and plate count agar supplemented with 0.1% milk and 1% salt (PCAMS; designed for cheese rind bacteria) (22) and incubated them for up to 5 days to maximize recovery. DNA sequencing of these cultured bacterial communities, of the near-full-length 16S rRNA gene using Nanopore-based sequencing, generated 171,926 high-quality reads, grouped into 36 OTUs, which belong to five phyla, seven classes, 14 orders, 25 families, and 36 genera. Compared with our analysis of wood grindings, this sequencing was comparable in quality but revealed a much-reduced diversity, which could be partially due to our incomplete method of bacterial recovery.

Our analysis of live bacterial communities revealed that they share many common members with the total DNA from wood grindings (Fig. S1). Most notably, *Staphylococcus* was the most dominant bacterium, consistently making up 10%–98% of the cultured communities across all boards. *Brevibacterium* and *Brachybacterium* were also universally present, although their abundance varied significantly. *Brevibacterium* was found at 0.06%–8%, whereas *Brachybacterium* accounted for 0.04%–85% of the communities. In addition to these core members, several other salt-tolerant bacteria were found in relatively high numbers within the active, culturable communities. These included *Psychrobacter* (up to 33.6%), *Virgibacillus* (up to 16%), *Halomonas* (up to 5%), *Vibrio* (up to 3%), *Oceanobacillus* (up to 3.4%), and *Cobetia* (up to 2.3%). Importantly, we also identified an enrichment of certain bacteria whose abundances were too low in the total DNA composition of wood grindings. These included *Bacillus*, *Lactococcus*, and *Vibrio*, as well as *Mammaliicoccus* (related to *Staphylococcus*) and *Niallia* (related to *Bacillus*). Interestingly, sanitized boards also harbored many bacteria that were dominant on native boards, especially *Staphylococcus*, *Brevibacterium*, and *Brachybacterium* (Fig. S1). Furthermore, the sanitized board from cheese maker 1 harbored somewhat similar communities to native boards, suggesting that these bacteria are part of the facility microbiome (Fig. S2).

### *L. monocytogenes* declines in the clean zones of most wooden cheese boards

A previous study showed *L. monocytogenes* to be killed on the surface of native wooden boards (19). However, recovery of *L. monocytogenes* from wood is notoriously poor and could confound quantification of *L. monocytogenes* burdens on wood under prolonged incubation. We therefore examined both *L. monocytogenes* burdens and native cheese board bacteria as controls for our recovery method and incubation conditions.

**Fig 3:**
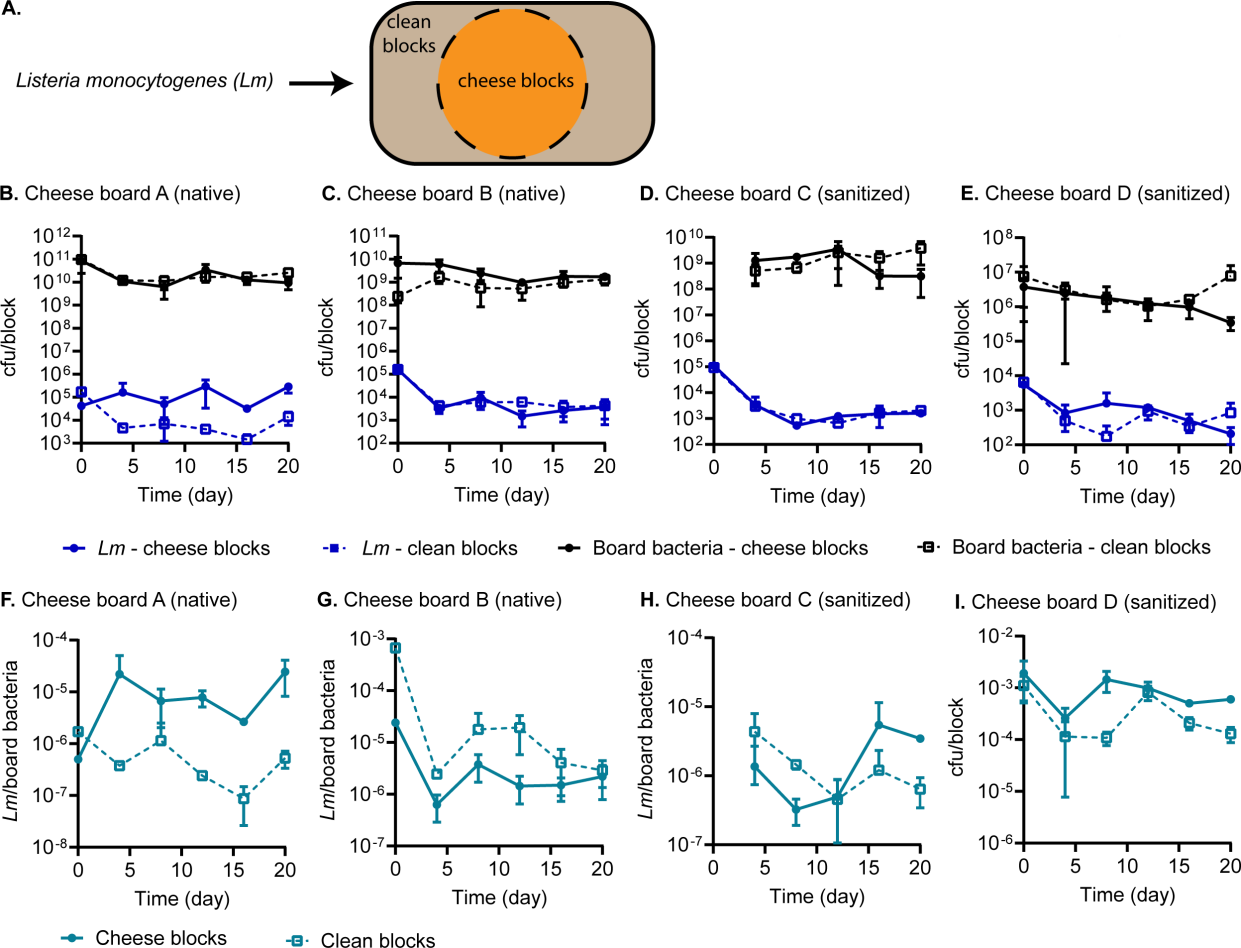
*L. monocytogenes* generally decline on wooden cheese boards following surface inoculation. (**A**) Schematic diagram of a wooden board with clean and cheese blocks, which were separated prior to surface inoculation with *L. monocytogenes*. Boards A and B are equivalent to boards 2 and 3, and boards (**C and D**) are equivalent to board 1 in Fig. 1 and 2. (**B–E**). Burdens of *L. monocytogenes* and board-associated bacteria on each block. (**F–I**) Relative burdens of *L. monocytogenes*, normalized to those of board-associated bacteria from each block. Native board-associated bacteria were not collected from board C at time point 0.

We obtained four boards from cheese maker 1, including two native boards similar to boards 2 and 3, and two sanitized boards similar to board 1, in our microbiota analyses. We again separated clean blocks from those inside the cheese zone because cheese residues can impact *L. monocytogenes* growth and survival (Fig. 3A). Each board was cut into square blocks, surface inoculated with *L. monocytogenes,* and incubated at 12°C, 85% relative humidity for 20 days. To recover bacteria from those blocks, we applied a destructive method, shown to have the highest recovery rate, which involved shaving, grinding, and rigorous suspension in phosphate-buffered saline.

Over 20 days, board-associated bacterial counts remained steady (Fig. 3B through E). The native boards harbored ~10^9^–10^10^ cfu/block (~1.7 × 10^7^–1.7 × 10^8^ cfu/cm^2^), whereas the sanitized boards harbored ~2 × 10^6^–10^9^ cfu/block (~1.7 × 10^4^–1.7 × 10^7^ cfu/cm^2^). Examining raw counts of *L. monocytogenes* on wooden blocks, we found them to generally decline over the incubation period. The reduction in *L. monocytogenes* was most evident on clean blocks, with a ~1.5 log cfu decrease over the first 4 days, and a similar magnitude of reduction was also apparent on cheese blocks of boards B, C, and D. The only exception to this trend was on cheese blocks of board A, where *L. monocytogenes* burdens exhibited a modest increase on day 20 compared with day 0 (Fig. 3B through E).

Upon normalizing *L. monocytogenes* counts to those of board-associated bacteria on each block, we observed an apparent increase in the relative abundances of *L. monocytogenes* on cheese blocks of board A, with a nearly 2-log increase in cfu counts between days 0 and 20 (Fig. 3F through I). However, *L. monocytogenes* burdens otherwise declined on cheese blocks of other boards and on clean blocks of all boards. Clean blocks of board B were the most inhibitory, causing a ~2 log cfu reduction in *L. monocytogenes* over 20 days. The magnitude of *L. monocytogenes* decline on other blocks was ~0.5- and 1-log cfu.

### The wooden board microbiota harbor bacteria that inhibit *L. monocytogenes*

A previous study identified *Leuconostoc mesenteroides* and *Staphylococcus equorum*, isolated from wooden boards, to inhibit the growth of *L. monocytogenes* (21). The microbial diversity of wooden boards in our study suggests that there might be more bacterial species from these boards that antagonize *L. monocytogenes*. Therefore, we systematically isolated cheese board-associated bacteria by recovering wood chip suspensions in different growth media, including TSA, Brain Heart Infusion (BHI) agar, De Man–Rogosa–Sharpe agar (MRS), and PCAMS (22). This effort yielded approximately 500 bacterial isolates that were screened for antimicrobial activity by spotting on a *L. monocytogenes* lawn on suitable agar. Around 220 isolates produced a zone of clearance, indicating antimicrobial activity, and these were further purified through multiple rounds.

**Fig 4:**
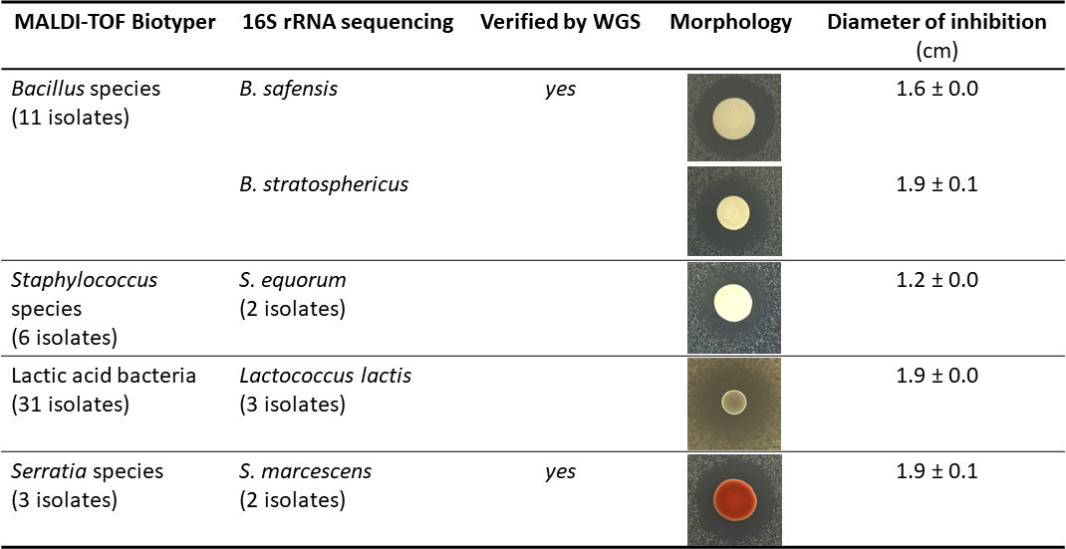
Identification of antimicrobial-producing bacteria from wooden cheese boards. Bacterial isolates that inhibited *L. monocytogenes* were initially identified by MALDI-TOF. A subset of those isolates was identified by Sanger sequencing of the 16S rRNA gene, and by whole genome sequencing as indicated. Inhibition of *L. monocytogenes* lawns was tested on 50% BHI agar and MRS agar (for *Lactococcus lactis*). Pictures of inhibition zones are not scaled.

Through the above culture-based method, we obtained 54 bacterial isolates representing four major types of colony morphology. Identification by a MALDI-TOF Biotyper revealed that those isolates belong to *Bacillus*, *Staphylococcus*, *Lactococcus*, and *Serratia* genera, although the species could not be unambiguously identified. Therefore, we selected nine isolates for 16S rRNA sequencing and identified them as shown in Fig. 4. These bacteria include two *Bacillus* species, many isolates of *Staphylococcus equorum*, *Lactococcus lactis*, and *Serratia marcescens*. The bactericidal effect of *L. lactis* is most likely due to bacteriocins, which are well-studied (23). Inhibition of *L. monocytogenes* by other bacteria could be due to secreted organic acids. To assess this, we measured pH values of their culture supernatants and found them to be mildly acidic or basic, ranging from pH 6.5 to 8.5 (Fig. S3A). In BHI broth adjusted to these pH values, *L. monocytogenes* growth was modestly reduced or unaffected (Fig. S3B and C). Therefore, *L. monocytogenes* killing was not due to the effects of pH alone, although it remains possible that these bacteria produce organic acids that exert membrane stress, independent of acid stress. Furthermore, several of these bacteria are known to produce diffusible antimicrobials. *Staphylococcus equorum* secretes micrococcin P1, which is bacteriostatic (21, 24), and *S. marcescens* produces a serrawettin lipopeptide that is bactericidal (25).

### *Bacillus safensis* secretes antimicrobials against *L. monocytogenes*

*B. safensis* and *B. stratosphericus* were the most potent and consistent inhibitors of *L. monocytogenes* in our screen (Fig. 4). We chose to focus on *B. safensis*, an environmental bacterium of soil origin with antifungal activity (26).

**Fig 5:**
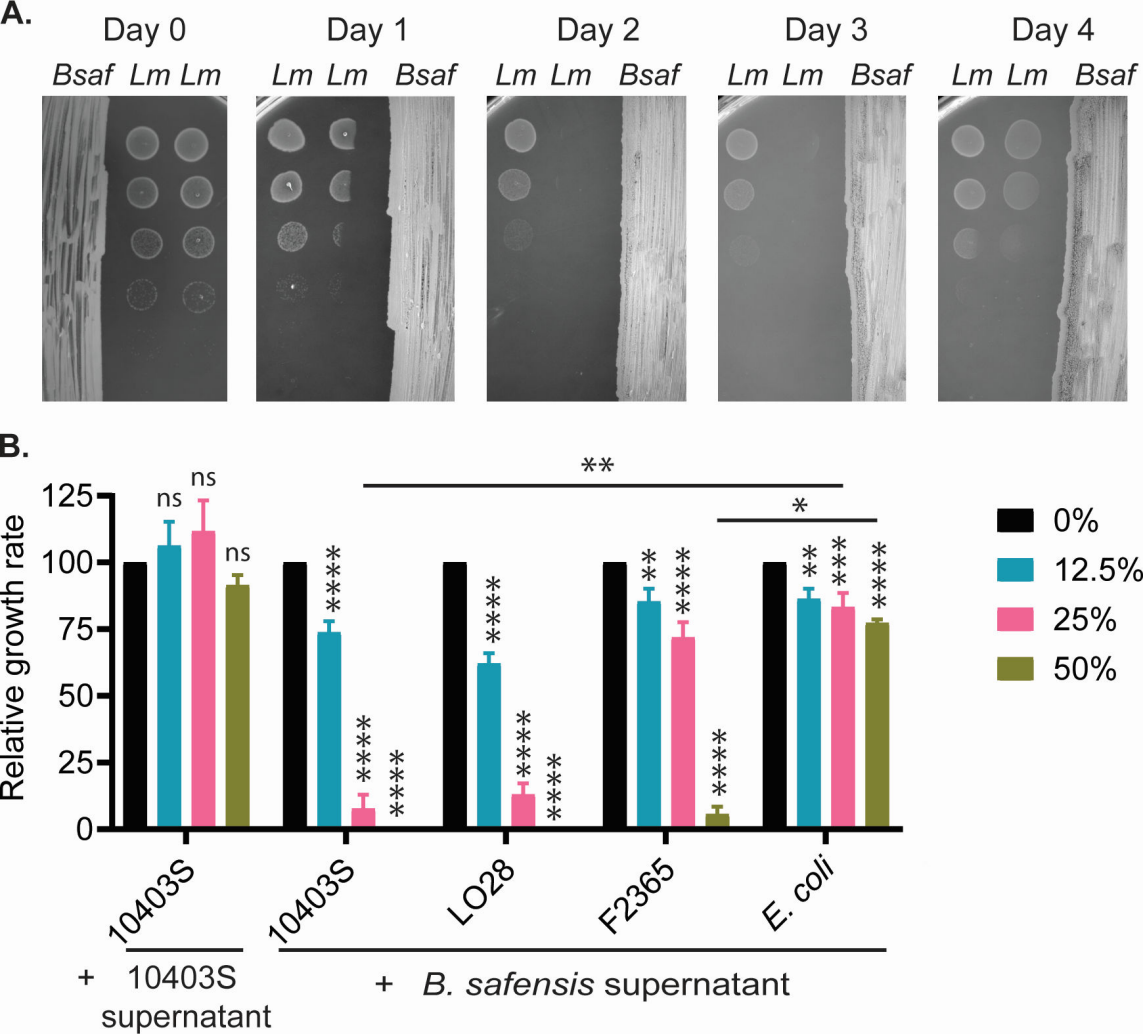
*B. safensis* inhibits *L. monocytogenes* growth via secreted antimicrobial factors. (**A**) Inhibition of *L. monocytogenes* growth on BHI agar. *B. safensis* was streaked on one half of the agar and pre-grown for 0– 4 days at 30°C. Tenfold dilutions of *L. monocytogenes* cultures were spotted on the other half of the agar, on the indicated number of days after *B. safensis* was streaked. (**B**) Inhibition of *L. monocytogenes* growth in BHI broth at varying concentrations of *B. safensis* supernatant. Relative growth rate was calculated as the growth rate in the presence of added supernatant, normalized to that in BHI broth only. Statistics: one-way ANOVA within each strain, and two-way ANOVA among strains. ns: non-significant; *, *P* < 0.05; **, *P* < 0.01; ***, *P* < 0.001; ****, *P* < 0.0001.

The zone of *L. monocytogenes* clearance surrounding *B. safensis* growth suggests that *B. safensis* might produce and secrete antimicrobials against *L. monocytogenes*. To further test this possibility, we plated serial dilutions of *L. monocytogenes* away from *B. safensis* on an agar medium. *L. monocytogenes* was indeed inhibited by *B. safensis* despite spatial separation, confirming that *B. safensis* secretes anti-*Listeria* factors (Fig. 5A). In this experimental setup, antimicrobial synthesis and secretion were somewhat delayed, since *L. monocytogenes* was only inhibited if *B. safensis* was pre-grown for at least 1 day prior to *L. monocytogenes* plating. The inhibitory effect was diminished by 4-day-old *B. safensis*, suggesting that secreted antimicrobials were unstable by this time point. Together, these data also reveal that *B. safensis*-derived antimicrobials accumulate as the culture grows, and their synthesis is not triggered by *L. monocytogenes*.

To evaluate secreted antimicrobials in liquid cultures, we obtained a cell-free culture supernatant from *B. safensis* that had been grown to a high density (OD_600_ > 10) and tested it against *L. monocytogenes* growth. As controls, we also obtained *L. monocytogenes* culture supernatants from the same culture condition. We found *L. monocytogenes* to be potently inhibited by supernatant from *B. safensis*, but not *L. monocytogenes* (Fig. 5B). Furthermore, *E. coli* MG1655 was not inhibited by *B. safensis* supernatant. This observation indicates that *L. monocytogenes* inhibition likely occurs through antimicrobial activity of *B. safensis* rather than nutrient depletion in spent media.

Our assessments of *L. monocytogenes* inhibition thus far have focused on strain 10403S (sequence type 85). Because *L. monocytogenes* is genetically diverse, we expanded our analysis to include other commonly studied strains: F2365 (sequence type 1), EGD (sequence type 12), EGD-e (sequence type 35), and LO28 (sequence type 210) (27, 28). Strain F2365 was the most resistant, but all strains were completely inhibited by 50% *B. safensis* supernatant (Fig. 5B).

### Cheese board-associated *B. safensis* encodes many biosynthetic gene clusters with predicted antimicrobial functions

**Fig 6:**
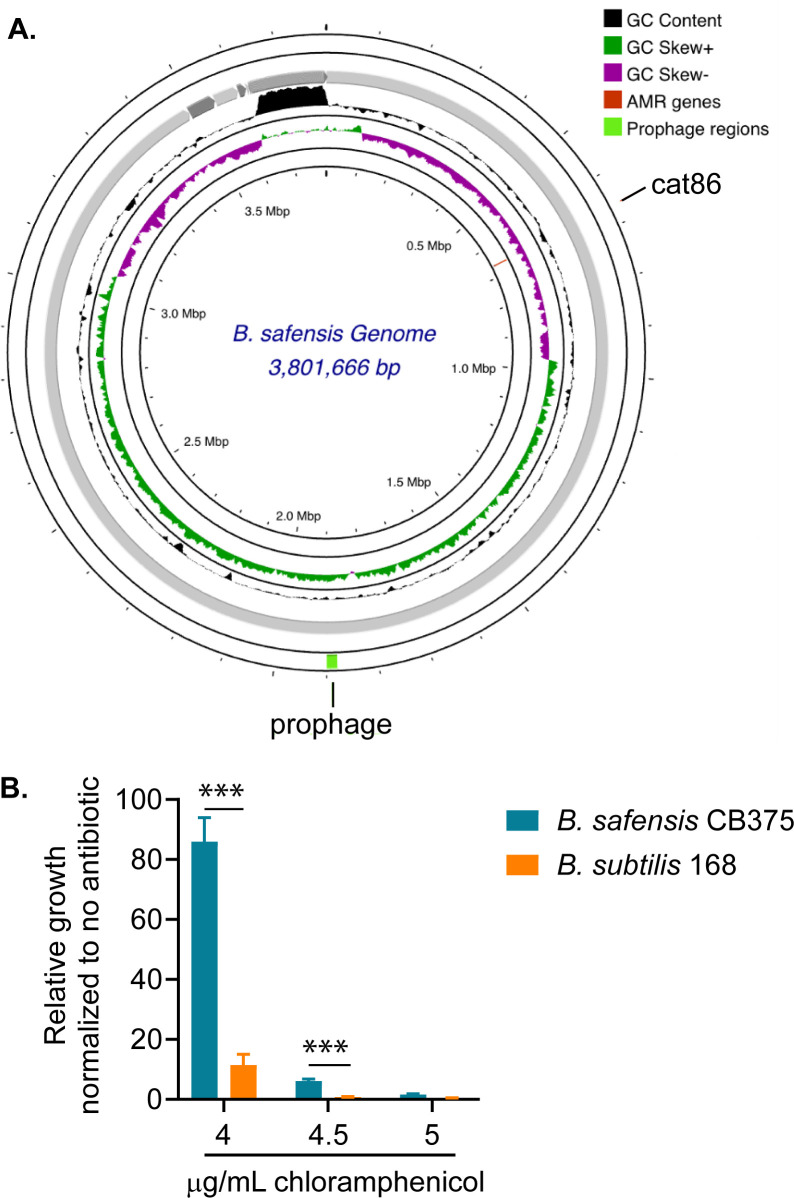
Genomic analysis of *B. safensis* strain CB375. (**A**) The assembled *B. safensis* CB375 genome with a predicted chloramphenicol acetyltransferase gene (*cat86*). (**B**) Chloramphenicol resistance of *B. safensis* CB375 and *B. subtilis* 168, grown in LB at 37°C. For each bacterium, growth at each chloramphenicol concentration was normalized to growth in LB only. Statistical analysis was performed by Student’s for the indicated pairs: ***, *P* < 0.001.

As a first step toward developing *B. safensis* for antimicrobial production against *L. monocytogenes*, we performed whole genome sequencing to gain genetic knowledge for the isolate obtained in this study. Our cheese board *B. safensis* isolate, hereafter called CB375, has a genome of approximately 3.8 Mbp with 41.64% GC content, harboring 3,763 genes, encoding 3,692 proteins, 64 tRNA genes, and 6 rRNA genes (Fig. 6A). Of the 3,781 CDS predicted, 1,184 are proteins of unknown function, and 2,579 proteins have functional assignments, including 1,312 proteins with Enzyme Commission (EC) numbers, 1,799 with Gene Ontology (GO) assignments, and 752 proteins that can be mapped to Kyoto Encyclopedia of Genes and Genomes (KEGG) pathways. By phylogenetic and average nucleotide identity (ANI) analyses, we confirmed that CB375 is *B. safensis* and most closely related to strain FUA2118 (Fig. S4; Table S1). Antimicrobial resistance gene detection by the CARD database (29) indicated that *B. safensis* CB375 harbors a putative chloramphenicol acetyltransferase (CAT), which exhibits 42% identity and 66% similarity to CAT from commonly used bacterial expression plasmids (Fig. S5). In LB broth, *B. safensis* CB375 was noticeably more chloramphenicol resistant than *B. subtilis* type strain 168, which does not carry a *cat* gene, although their minimum inhibitory concentrations were similar: approximately 5 µg/mL for *B. safensis* and 4.5 µg/mL for *B. subtilis* (Fig. 6B).

*Bacillus* species produce a wide range of antimicrobial peptides, encoded by biosynthetic gene clusters (BGCs). We therefore mined the *B. safensis* CB375 genome for BGCs using the antiSMASH database. This analysis revealed 14 BGCs, most of which are predicted to encode non-ribosomal peptide synthases (NPRS) (Table 1; Fig. S6). Four BGCs within *B. safensis* CB375 are highly similar to those producing known compounds. BGC5 perfectly matches the reference BGC for bacillibactin, a siderophore that has antibacterial and antifungal activities due to iron sequestration (30). BGC2 is 91% similar to plantazolicin, a peptide of the RiPP family (ribosomally synthesized and post-translationally modified peptides) with a narrow spectrum activity towards *B. anthracis*, *B. cereus*, and *B. thuringiensis* (31). BGC6 and BGC12 exhibit 85% similarity to bacilysin and sporulation killing factor, respectively (32–35). Five BGCs have low similarity to fengycin (an antifungal peptide), schizokinen (a siderophore), lichenysin, and sporulation killing factor (36, 37). Although BGC14 is predicted to produce lichenysin, it is divergent from the reference BGC and most likely synthesizes a different compound (Fig. S6). The remaining five BGCs do not match with reference BGCs and are unique to *B. safensis* and closely related species (Table 1).

**TABLE 1:** Predicted biosynthetic gene clusters within *B. safensis* CB735

BGC no.	Annotation	Similar known BGC	Reference organism
1	Betalactone	Fengycin 53% similarity to BGC0001095	*B. velezensis*
2	LAP;RRE-containing	Plantazolicin 91% similarity to BGC0001173	*B. pumilus*
3	NI-siderophore;terpene	Schizokinen 62% similarity to BGC0002633	*Leptolyngbya* sp.
4	RRE-containing	No matches found	
5	NRP-metallophore;NRPS	Bacillibactin 100% similarity to BGC0000309	*B. subtilis*
6	Other	Bacilysin 85% similarity to BGC0001184	*B. velezensis*
7	Betalactone	No matches found	
8	RiPP-like	No matches found	
9	T3PKS	No matches found	
10	Terpene	No matches found	
11	NRPS	Lichenysin 50% similarity to BGC0000381	*B. licheniformis*
12	Sactipeptide	Sporulation killing factor 85% similarity to BGC0000601	*B. subtilis*
13	NRPS	Surfactin 43% similarity to BGC0000433	*B. velezensis*
14	NRPS	Lichenysin 14% similarity to BGC0000381	*B. licheniformis*

### *B. safensis*-derived antimicrobials that inhibit *L. monocytogenes* are peptides or proteins

**Fig 7:**
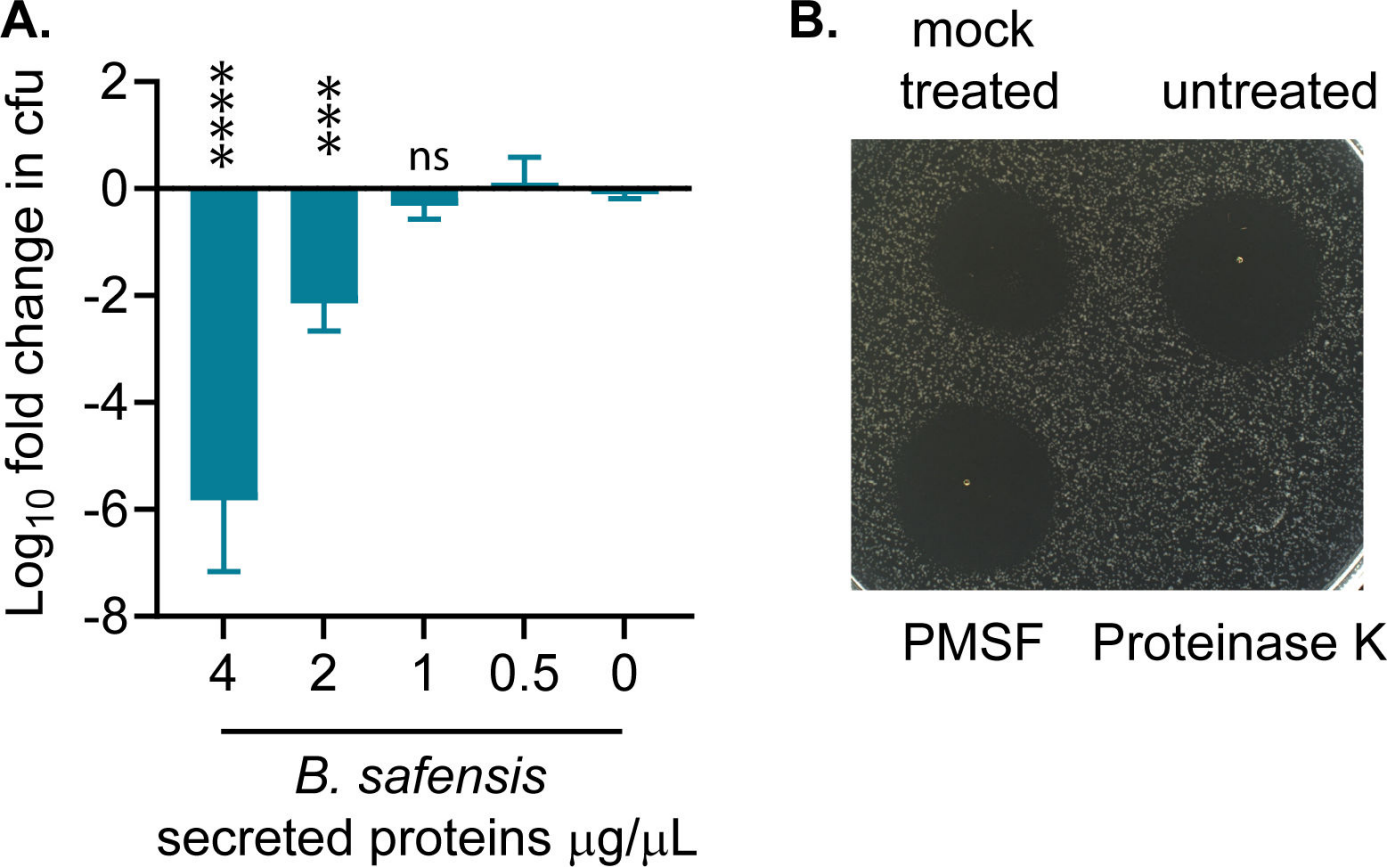
*B. safensis*-secreted antimicrobials against *L. monocytogenes* are proteins or peptides. (**A**) Killing of *L. monocytogenes* by concentrated compounds from *B. safensis* culture supernatants. *B. safensis* supernatants were concentrated with ammonium sulfate and resuspended in PBS, then used to treat *L. monocytogenes* cultures, at approximately 10^9^ cfu/mL, for an hour. Statistical analysis was performed by one-way ANOVA, comparing each treatment with the control. ****, *P* < 0.0001; ***, *P* < 0.001; ns: non-significant. (**B**) Concentrated *B. safensis* supernatants were treated with proteinase K for 2 h at 37°C, 0.5 mM PMSF (phenylmethylsulfonyl fluoride), or mock-treated, and spotted on a *L. monocytogenes* lawn to inspect for antimicrobial activities.

Given the presence of many antimicrobial peptide-encoding BGCs within the *B. safensis* genome, we sought to isolate secreted peptides from *B. safensis* by ammonium sulfate precipitation of its culture supernatant. These concentrated preparations of secreted compounds were bactericidal toward *L. monocytogenes* in a dose-dependent manner, with up to ~6 log cfu of killing after 1 h of exposure (Fig. 7A). Furthermore, proteinase K treatment abolished *L. monocytogenes* inhibition, further verifying that antimicrobial compounds are proteins or peptides in nature (Fig. 7B). Combined with the predicted presence of multiple BGCs, *B. safensis* CB375 most likely inhibits *L. monocytogenes* through secreted antimicrobial peptides.

### *B. safensis* supernatant induces a specific stress response by *L. monocytogenes*

**Fig 8:**
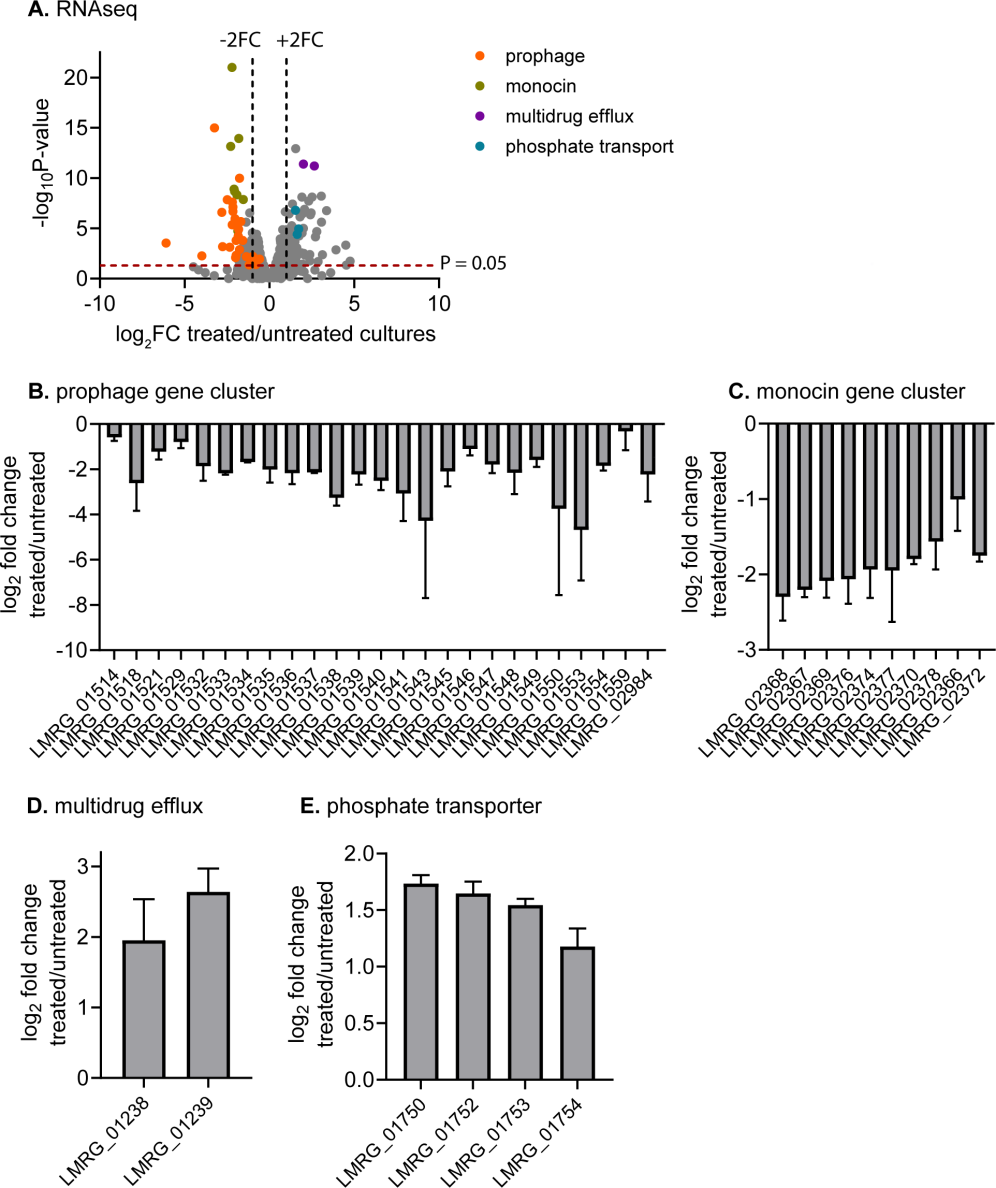
Transcriptional response of *L. monocytogenes* to *B. safensis* cell-free culture supernatant. (**A**) Volcano plot depicts *L. monocytogenes* genes that were up- and down-regulated during growth in 1.5% *B. safensis* supernatant, compared with untreated cultures. (**B–E**) Notable down- and upregulated genes in treated vs. untreated *L. monocytogenes* cultures. Log_2_ fold change in gene expression was calculated by EdgeR, using normalized read counts per million.

Although *L. monocytogenes* was potently inhibited by *B. safensis* culture supernatants and concentrated peptide preparations, *Bacillus* species were not abundant in the surface microbiota of wooden boards in our study. We therefore examined the response of *L. monocytogenes* to a low dose of *B. safensis* culture supernatant (1.5%) that modestly inhibited *L. monocytogenes* growth (Fig. S7). Using a stringent fold change cutoff, we found this treatment to significantly upregulate 25 genes by ≥4-fold and significantly downregulate 16 genes by ≥4-fold compared with untreated *L. monocytogenes* cultures (Fig. 8). Strikingly, all downregulated genes map to the Φ10403S prophage or monocin locus (38). However, comparing two isogenic *L. monocytogenes* strains with and without the Φ10403S prophage, we found them to be equally sensitive to *B. safensis* (Fig. S8). The upregulated genes included a drug efflux system (*lmo2087-lmo2088*), consistent with an antimicrobial stress response. Furthermore, a putative phosphate transport system, homologous to the high-affinity PstSCAB phosphate transporter (39), and an operon of unknown function, *lmo2567-lmo2568*, were also significantly upregulated.

## DISCUSSION

With a goal to expand our knowledge of wooden cheese board microbiota, we conducted a small survey of seven wooden boards from three different cheese makers in Wisconsin, United States. Although we expected an overlap between the microbiota of cheese rind and wooden boards, we hypothesized that wood harbors native bacteria that are not directly transferred from cheese. To analyze the microbiota that reside on board surfaces during storage, we obtained sanitized boards and native boards at the completion of ripening. Our analysis of total microbial DNA confirms the diversity of bacterial communities on wooden boards in cheese ripening facilities. Of the 93 genera identified on those boards, *Staphylococcus*, *Brevibacterium*, and *Brachybacterium* were universally present and were among the most abundant bacteria in those microbial communities. On clean zones of wooden boards outside of cheese blocks, these three genera comprised up to 36%, 60%, and 32% of the total bacterial abundance, respectively. Their abundance is congruent with two previous surveys of wooden boards in other locations (4, 5). Furthermore, we found these bacteria to also be prominent in board areas where cheese blocks had been placed, consistent with their ubiquitous presence on cheese rinds (7). All boards in our study also presented an abundance of *Nocardiopsis*, a genus of halotolerant bacteria that is also abundant in cheese rind communities, but not in other food products (7). Together, these observations suggest that *Staphylococcus*, *Brevibacterium*, and *Brachybacterium* are core members of wooden board microbial communities and highlight the reciprocal microbial transfer between wood and cheese surfaces.

Our principal component analysis indicated that bacterial signatures on clean and “cheese” zones were different on most boards, presenting an opportunity to distinguish cheese-associated bacteria from those from dairy processing environments. Upon further inspections, we observed a particularly high abundance of *Ruania* in clean zones of wooden boards from cheese maker 3 (up to 18% of bacterial communities), although their abundance was rather low in other cheese ripening facilities. Therefore, *Ruania* is likely associated with the ripening environment of cheese maker 3 and might have been deposited onto wooden boards from aerosols or splashing. More thorough analyses are necessary to distinguish environmental bacteria from those of cheese origins, and their relative contributions to the ripening process.

Analyses of microbial DNA from wood shavings offer a culture-independent, comprehensive survey of board microbiota. However, such an approach likely reports the cumulative microbial history, including nonviable microorganisms, and might not accurately reflect the live microorganisms that stably reside on board surfaces. Indeed, we frequently isolated *Bacillus* and *Lactococcus* species despite their extremely low abundances in the total bacterial DNA footprints on wooden boards and rarely encountered *Brevibacterium* that was expected to be abundant. Therefore, we also performed a preliminary survey of bacterial communities that can be recovered from wooden boards under laboratory conditions.

The live, culturable bacteria on those boards harbored many bacteria that were abundant in the microbiota composition of wood grindings, consistent with a previous observation that cheese rind bacteria are mostly culturable (7, 22). Although our recovery method of live bacteria was not exhaustive, we identified many live bacteria that were not detected in analyses of wood shavings, likely due to their low abundances in the total microbial footprints. As an example, *Bacillus* species were consistently present on boards, although they are not typically associated with cheese rind. A more thorough recovery approach will likely uncover more environmental bacteria that colonize wooden board surfaces, and those bacteria might explain variations in flavor profiles among cheese production facilities.

We compared the bacterial compositions on native and sanitized boards as an additional approach to identify native bacterial communities that stably reside on board surfaces. As expected, sanitized boards in our study were not sterile, but they interestingly harbored many bacteria that were also abundant on native, unsanitized boards. This observation suggests that cheese ripening facilities and wooden boards are also their natural habitats. Larger surveys are necessary to establish the origins and stability of resident board communities.

Our study also sought to assess *L. monocytogenes* survival on wooden cheese boards, with three technical improvements compared with a previous study (19). First, we applied grinding, a disruptive method, to maximize *L. monocytogenes* recovery from wood following surface inoculation (40). Second, recognizing that even the most effective method only recovers less than 30% of inoculated bacteria on wood, we normalized *L. monocytogenes* burdens to native board bacteria recovered from wooden boards. Raw *L. monocytogenes* counts on each board suggested that *L. monocytogenes* was inhibited. However, normalized burdens revealed one instance of an increase in relative abundance of *L. monocytogenes*, by 2-log in the “cheese” zone of board A. Therefore, internal standards are necessary controls for the quantification of pathogens from wood. Finally, our study tracked *L. monocytogenes* burdens for 20 days following surface inoculation, much longer than a previous study (19), thereby providing safety data for longer storage of wooden boards.

Although *L. monocytogenes* grew within the cheese zone of board A, our aggregated data suggest that board wood inhibits *L. monocytogenes*. First, *L. monocytogenes* burdens declined in the clean area of board A, outside of the cheese block. Second, a reduction in *L. monocytogenes* burdens, by 0.5- to 2-log cfu, also occurred on other boards, both in the cheese and clean zones. Of note, board A was used for ripening of a soft cheese, which might explain its permissiveness for *L. monocytogenes* growth where there was leftover cheese. Combining the stability of native bacterial populations and the overall decline in *L. monocytogenes* counts, our data suggest that wooden boards are resilient to pathogen contamination over long-term storage. Analyses of additional boards over extended periods will be necessary to identify the core antimicrobial communities on wooden boards.

The inhibition of *L. monocytogenes* on wooden boards can be attributed to antimicrobial activities of both wood and the resident microbiota. Trees naturally produce antimicrobial compounds as a defense against pathogens, and essential oils or polyphenolic compounds from wood have been demonstrated to effectively kill foodborne pathogens (20). However, since those studies mainly examined pathogen survival in highly concentrated antimicrobial extracts, the extent and kinetics of pathogen killing on cheeseboard wood remain to be determined. Within the wooden board microbiota, several prominent bacteria are known to produce antimicrobial compounds. For instance, *L. monocytogenes* is inhibited by various bacteriocins from lactic acid bacteria (23), phenazines and antimicrobial peptides from *Brevibacterium linens* (41, 42), and micrococcin P1 from *Staphylococcus equorum* (24). By systematically screening culturable bacteria from wooden cheese boards, we identified additional antimicrobial-producing bacteria whose abundances were low, such as *Serratia marcescens* and *Bacillus* species.

We focused on characterizing a wooden board *B. safensis* isolate, CB375, as a novel candidate for antimicrobial discovery against *L. monocytogenes. B. safensis* is a soil-dwelling bacterium that can colonize plant roots and stimulate plant growth (43–45), and some *B. safensis* strains have potent antifungal activity (26). Here, we found that *B. safensis* CB375 inhibits *L. monocytogenes* via secreted compounds that can be easily obtained and concentrated in the culture supernatant. Our bioinformatics analysis detected 14 biosynthetic gene clusters that encode antimicrobial peptides within the *B. safensis* CB375 genome. Six of those BGCs have no known homologs and likely encode novel compounds for future antimicrobial discovery efforts. Seven BGCs are highly similar, both in gene cluster organization and sequences, to those for known compounds, including fengycins, plantazolicin, schizokinen, bacillibactin, bacilysin, lichenysin, and sporulation killing factor. Fengycins are amphiphilic cyclic lipopeptides that are fungicidal against filamentous fungi through membrane permeabilization (36, 46). Plantazolicin has a narrow-spectrum activity toward *B. anthracis*, *B. cereus*, and *B. thuringiensis* and is therefore unlikely to inhibit *L. monocytogenes* (31). Schizokinen and bacillibactin are siderophores that can help *Bacillus* species in competitions with other bacteria (47), such as the inhibition of *Pseudomonas* by bacillibactin (30, 48), but their effect against *L. monocytogenes* has not been tested. Bacilysin from *B. subtilis* is important for cellular differentiation and can inhibit other bacteria, such as *Campylobacter jejuni* (34, 49), but its antimicrobial activity has not been broadly characterized. Lichenysin is a lipopeptide biosurfactant that can disperse bacterial biofilm, but there is yet no evidence for direct antibacterial activities (50, 51). Similarly, sporulation killing factors lyse kin cells within a *Bacillus* species population as a survival strategy under carbon source starvation and are unlikely to act against other species (36, 37). Finally, BGC13 within *B. safensis* CB375 harbors several genes similar to a surfactin-encoding reference BGC, but with substantial differences in genetic organization. Therefore, BGC13 likely encodes a different surfactin homolog. Surfactins, widely produced by many *Bacillus* species, are membrane-permeabilizing cyclic lipopeptides that can inhibit different bacteria (52, 53). However, more genetic analyses are needed to establish if their activities are physiologically significant in microbial communities (53). A recent study identified safencin E as a novel class II bacteriocin from a *B. safensis* isolate from bees’ gut (54). Although class II bacteriocins generally have broad-spectrum activity (55), the antimicrobial effect of safencin E toward *L. monocytogenes* remains to be tested. Nevertheless, the *B. safensis* CB375 genome does not harbor a homologous BGC for safencin E.

*L. monocytogenes* strain 10403S encodes two phage-like elements, a Φ10403S prophage and a monocin, that are predicted to attack other bacteria (38). Both elements were significantly downregulated in response to a sub-inhibitory concentration of *B. safensis* supernatant. As for other bacteria, prophage and monocin gene expression in *L. monocytogenes* is induced by the SOS response and activated upon treatment with DNA-damaging reagents (38). The expression of these loci is also inhibited by prophage-encoded AriS, which suppresses the SOS response, and derepressed by monocin-encoded MpaR (38, 56). Therefore, the repression of prophage and monocin suggests that *B. safensis*-derived antimicrobial peptides might activate AriS, inhibit MpaR, or suppress SOS response by other unknown mechanisms. Among upregulated genes, an increased expression of drug efflux is consistent with an antimicrobial stress response. The upregulated *lmo2567-lmo2568* operon is homologous with the PstSCAB phosphate transporter (39), suggesting that a phosphate starvation response might be induced. However, the role of Lmo2567-Lmo2568 in phosphate transport and the impact of phosphate on *L. monocytogenes* antimicrobial response both need to be experimentally determined.

In summary, our study expands our knowledge of the microbial ecology on wooden boards used in cheese ripening. Our findings indicate an inhibitory effect of clean wooden boards against surface-inoculated *L. monocytogenes*. The relative contributions of wood and the resident microbiota to pathogen inhibition are unknown, but our identification of several bacteria that inhibit *L. monocytogenes* highlights wooden board microbiota as a source for antimicrobial discovery.

## MATERIALS AND METHODS

### Wooden boards

For microbiota analyses, seven wooden boards were obtained from three cheese makers in Wisconsin (United States), including native boards at the completion of cheese ripening or sanitized boards. Boards were sanitized in four steps: washing in a diluted detergent at ~75°C, rinsing with fresh water, dipping in a sanitizing reagent (peracetic acid), and air drying. Boards were numbered in this report as follows. All boards from cheese maker 1 were made of cedar: board 1 (sanitized), board 2 (native, used for a smear-ripened cheese), and board 3 (native, used for a hard cheese). Boards from cheese maker 2 were made of pine: board 4 (native, used for a hard cheese) and board 5 (sanitized). Boards from cheese maker 3 were made of pine: board 6 (native) and board 7 (used for a hard cheese).

For *L. monocytogenes* survival analyses, boards A–D were obtained from cheese maker 1. Board A was equivalent to board 2 (native board from the same smear-ripened cheese), board B was equivalent to board 3 (native board from the same hard cheese), and boards C and D were equivalent to board 1 (sanitized).

### Extraction of genomic DNA and sequencing of bacterial amplicons

Genomic DNA (gDNA) extraction of microbial community on the wooden board surfaces was performed based on a published procedure (4). Wood dust, obtained by drilling and shaving, was thoroughly resuspended in phosphate-buffered saline (PBS) by gentle rotation, pelleted by centrifugation, and mechanically disrupted by bead beating, with the addition of phenol and 20% sodium dodecyl sulfate. Lysates were then centrifuged, and DNA was extracted and purified using phenol:chloroform:isoamyl alcohol (25:24:1, pH 8) (Thermo Fisher Scientific), precipitated, and washed with ethanol. DNA samples were quantified with a Qubit fluorometer (Invitrogen).

Total genomic DNA samples were PCR amplified for the 16S rRNA V4 region using universal primers as previously published (57). Briefly, 50 ng of DNA was used as template in each reaction, amplified with HotStart Ready Mix (KAPA Biosystems). PCR products were gel-purified, quantified with a Qubit fluorometer, pooled in equimolar ratios, and sequenced on an Illumina MiSeq using a v2 kit to generate paired-end 250 bp reads with custom sequencing primers.

Sequences were demultiplexed on the Illumina MiSeq system and quality filtered using the q2-demux plugin, followed by denoising with DADA2 (58). Subsequent processing was performed using QIIME 2 (59). Amplicon sequence variants were aligned using mafft (via q2-alignment) (60) and used to construct a phylogeny with fasttree2 (via q2-phylogeny) (61). Taxonomy was assigned to ASVs using the q2-feature classifier. Relative abundances of the top 20 taxa among all the samples were plotted using phyloseq and fantaxtic R packages (62, 63).

### Recovery of bacteria from wooden boards and taxonomic identification

PBS suspensions of wood shavings obtained above were diluted and plated onto tryptic soy agar and PCAMS agar (22) and incubated at room temperature for up to 5 days. For each board, bacteria were pooled from all agar plates, and DNA was extracted using phenol:chloroform:isoamyl alcohol (25:24:1, pH 8) (Thermo Fisher Scientific), precipitated, and washed with ethanol. DNA samples were quantified with a Qubit fluorometer (Invitrogen).

Long-read Nanopore-based sequencing was used to characterize live microbes to the species level and thereby provide a more accurate census of their identities. Sequencing libraries were prepared from the gel-extracted 16S rRNA amplicon bands using the Rapid Barcoding Kit 96 V14 (Oxford Nanopore Technologies) and sequenced using PromethION flow cells (R10.4.1) on a P2 Solo device attached to a GridION (ONT) with MinKnow v 22.08.9. Super accurate base calling mode was selected along with barcode trimming. All other parameters were set to the default. Samples were base-called on the machine with the most up-to-date Dorado base caller.

For the full-length 16S rRNA gene amplicons, adapters were removed using Porechop (64), and sequences were filtered by size using Nanofilt (65), selecting for reads between 1,000 and 3,000 bp. Filtered fastq files were taxonomically classified using SINA (66), with the NR 99 138.2 SILVA database (67) as reference.

### Survival of *L. monocytogenes* on the wooden board surface

Wooden boards were cut into 24 blocks of 2 × 2 inches or 3 × 3 inches. Blocks from clean areas were separated from those inside the round area where cheese had been placed during ripening. *L. monocytogenes* 10403S (streptomycin resistant) was grown in Brain Heart Infusion broth, washed, resuspended in PBS, and spread evenly on each wooden board block. The blocks were placed in a closed container, maintained at 85% relative humidity using a saturated K_2_SO_4_ solution. All blocks were incubated at 12°C, and relative humidity was monitored using a digital hygrometer. For *L. monocytogenes* recovery at each time point, the surface was shaved off wooden blocks and ground into small bits inside a clean beaker. Bacterial counts on day 0 were obtained 30 min after surface inoculation to allow *L. monocytogenes* impregnation on wood. Wood shavings and bits were thoroughly resuspended in PBS, with gentle disruption using sterile glass beads. Serial dilutions were plated on BHI with 200 µg streptomycin to count for *L. monocytogenes* cfu, or BHI without antibiotics for total bacteria. Native board-associated bacterial counts were calculated as total bacterial counts subtracted by *L. monocytogenes* counts.

### Isolation and identification of wooden board bacteria that inhibit *L. monocytogenes*

Wooden boards were shaved and ground to obtain wood bits, which were thoroughly resuspended in PBS by gentle vortexing. The PBS slurries were plated onto the following agar media: BHI, TSB (Tryptic Soy Broth), PCAMS (22), and MRS. BHI, TSB, and PCAMS agar were incubated aerobically at 30°C. MRS agar was incubated at 37°C in a hypoxic chamber (4% CO_2_). Bacterial isolates were purified based on colony morphologies and spotted onto a lawn of 10^5^ cfu *L*. *monocytogenes*. Bacterial isolates that produced zones of clearance were purified and tested for at least four more rounds. Initial identification was achieved using a MALDI-TOF Bruker Biotyper at the Wisconsin Veterinary Diagnostic Laboratory. For further identification, bacterial DNA was PCR-amplified for the V4-V9 regions using primers 515F and 1492R as previously published (68). Bacterial species were identified using BLAST, with sequencing reads as the query.

### Whole genome sequencing and genomic analyses

Genomic DNA was extracted from *B. safensis* using phenol-chloroform (pH 8.0), precipitated and washed with ethanol, and resuspended in water. Illumina whole genome sequencing was performed at SeqCenter (Pittsburgh, PA, USA) to obtain 2 × 150 bp reads at a depth of 1.33 million reads per sample. Quality control and adapter trimming were performed with FastQC. Contig and genome assembly were performed *de novo* by SPAdes (69), with quality assessment by QUAST (70). BV-BRC web resources were used to confirm taxonomic identification, find similar genomes (about 100 genomes), and construct a phylogenetic tree. *B. safensis* CB375 and closely related genomes, revealed by the phylogenetic tree, were calculated for average nucleotide identity using KBase. The genome was annotated using Prokka (71), antimicrobial resistance genes were identified using CARD (29), and prophages were identified using Phigaro and VirSorter2 (72, 73). The genome map was generated with Proksee (74). Biosynthetic gene clusters (BGCs) were predicted using antiSMASH 6.0 (75) and zol (76).

### Inhibition and killing of *L. monocytogenes* by *B. safensis*

For inhibition on BHI agar, *B. safensis* was streaked on half of the petri dishes and pre-incubated for 0–4 days prior to spotting of serial dilutions of a *L. monocytogenes* culture in the other half of the petri dish. BHI agar was further incubated for another 16 h at 37°C to assess *L. monocytogenes* growth.

Cell-free culture supernatants were obtained from *B. safensis* cultures grown in BHI broth at 30°C for 16–20 h and filtered through a 0.22 µm PES membrane. As a control, cell-free culture supernatants were also obtained from *L. monocytogenes* cultures grown at 37°C in BHI broth. Inhibition of *L. monocytogenes* in BHI broth was assessed in 96-well plates containing BHI only or BHI containing indicated concentrations of cell-free culture supernatants. Bacterial growth was measured by OD_600_ at 37°C with intermittent shaking for 14 h in a plate reader. For each strain, growth rates at each supernatant concentration were normalized to the growth rate in BHI only of the same strain.

Secreted compounds from *B. safensis* were concentrated by adding a saturated ammonium sulfate solution (at a final 80%) to cell-free culture supernatants. Precipitates were separated by centrifugation and resuspended in PBS. Protein concentration was quantified by Bradford assay (Bio-Rad), using bovine serum albumin as a standard. For proteinase K treatment, proteinase K was added to concentrated protein/peptide preparations at a final concentration of 1 mg/mL, incubated at 37°C for 2 h, and 0.2 mM PMSF was added to inactivate proteinase K. As a control, 0.2 mM PMSF was added to another aliquot of concentrated proteins/peptides without proteinase K. For mock treatment, protein preparations were incubated at 37°C for 2 h.

For the assessment of *L. monocytogenes* killing, concentrated protein preparations from *B. safensis* culture supernatant were diluted to 4 µg/mL and added to *L. monocytogenes* cell suspensions in PBS. *L. monocytogenes* cfu were assessed at 0 and 1 h post-treatment, by plating serial dilutions on BHI agar + 200 µg/mL streptomycin.

### RNA-seq and data analysis

*L. monocytogenes* was grown in BHI to mid-log phase (OD_600_ ~0.5) without or with 1.5% *B. safensis* cell-free culture supernatant. RNA was extracted, and contaminated DNA was removed as previously described. RNA-seq was performed at SeqCenter (Pittsburgh, PA) to achieve 12 million 2 × 150 bp reads per sample. Reads were mapped to the *L. monocytogenes* 10403S genome (NCBI:txid393133). Differentially expressed gene analysis was performed by EdgeR (77).

## Data Availability

All 16S rRNA sequencing data were deposited in NCBI BioProject under accession number PRJNA1354512.

## SUPPLEMENTAL MATERIAL

Supplemental material spectrum.02936-25-s0001.docx Fig. S1 to S8; Table S1.

ASM does not own the copyrights to Supplemental Material that may be linked to, or accessed through, an article. The authors have granted ASM a non-exclusive, world-wide license to publish the Supplemental Material files. Please contact the corresponding author directly for reuse.
