# Supplementary material for: The microbiota of wooden cheese-ripening boards is a rich source of antimicrobial-producing bacteria against Listeria monocytogenes
Source: Microbiol Spectr. 2026 Jan 7;14(2):e02936-25. doi: 10.1128/spectrum.02936-25 (PMC12889095; doi:10.1128/spectrum.02936-25)
Supplement: Supplemental material — Fig. S1 to S8; Table S1. [file spectrum.02936-25-s0001.docx]

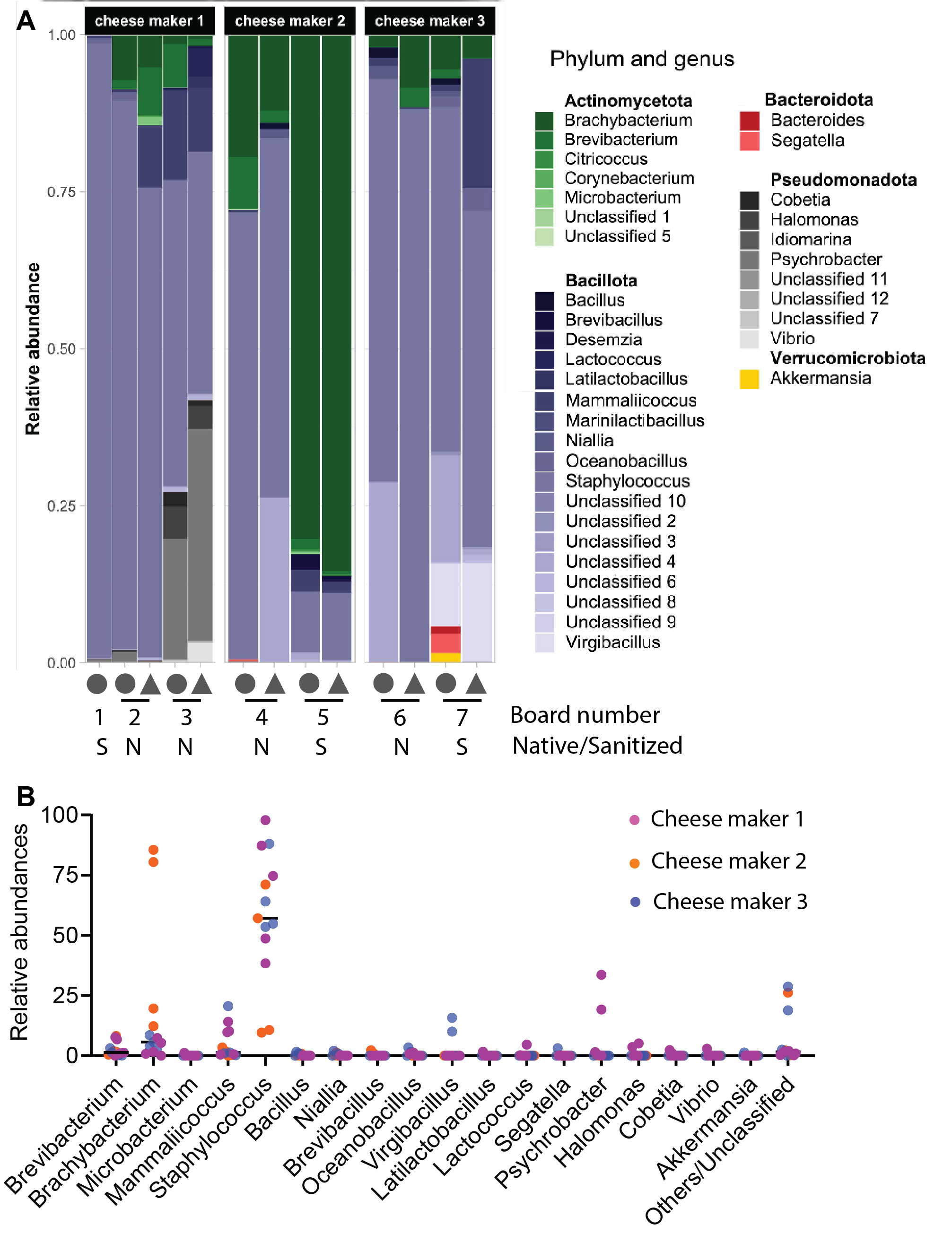


**Figure S1:** **Taxonomic survey of live bacteria recovered from wooden boards.** Bacteria from wooden cheese boards (same boards as Figures 1-2) were recovered on agar media, and DNA was pooled for taxonomic diversity. Relative abundances of the most abundant phyla on seven wooden boards are organized for each board (**A**) or each phylum (**B**). In panel A, circle symbol indicates cheese zone, and triangle indicates clean zone on each board. Bars in panel B are median abundances.


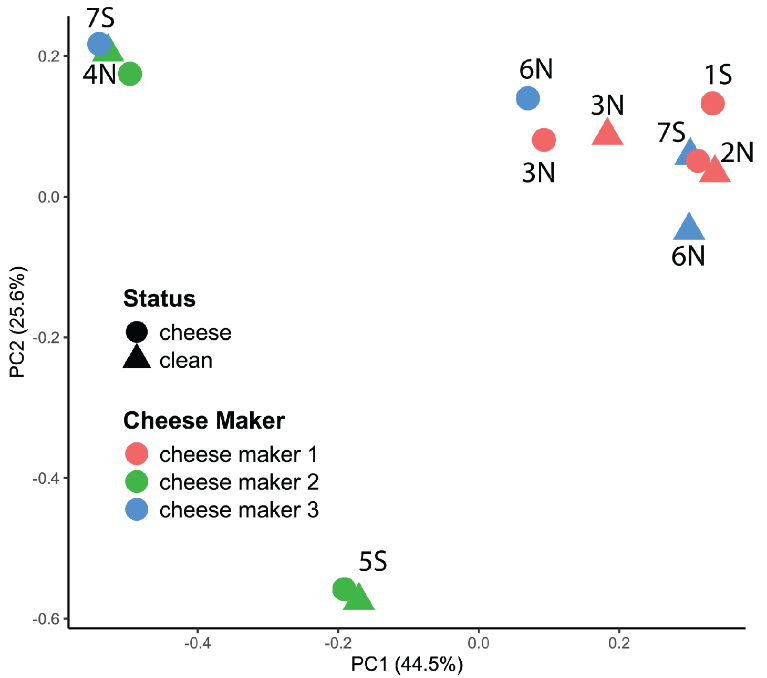


**Figure S2:** Principle component analysis of live bacteria recovered from wooden boards. These boards were the same as those analyzed in Figures 1-2. Each data point represents the bacterial composition of a native or sanitized wooden board from a cheese maker. Each board is marked with board number, N indicates native boards, S indicates sanitized boards.


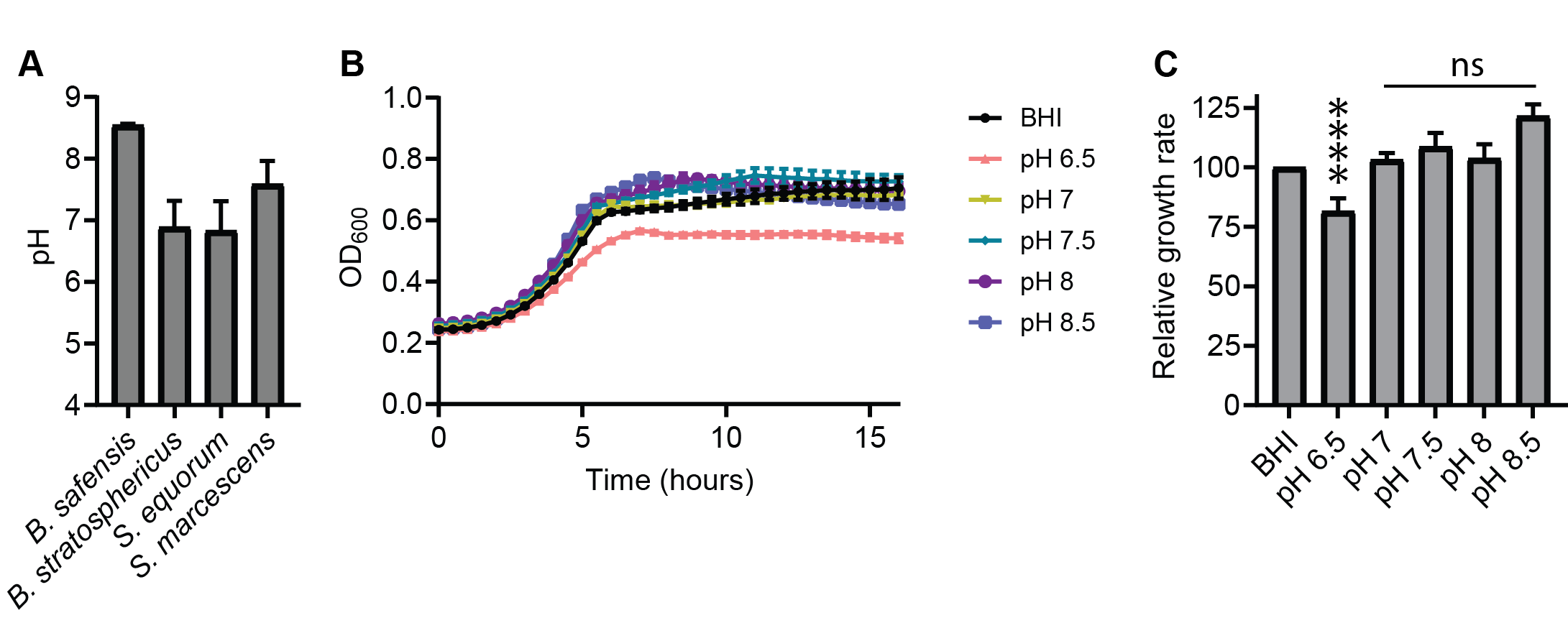


**Figure S3: *Listeria* growth at varying pH. A.** pH values of culture supernatants from bacteria that inhibit *L. monocytogenes*. All bacteria were grown in BHI at 37°C with shaking for 16 hours. **B.** Representative growth curves of *L. monocytogenes* in BHI broth, unadjusted, or adjusted to indicated pH values. Unadjusted BHI broth has a pH value of 7.3 ± 0.3 (average of 4 experiments). *L. monocytogenes* was grown at 37°C in a 96-well plate. **C.** Relative growth rates were calculated as exponential growth rates normalized to those in BHI broth only. Data in A and C are average of three independent experiments. Error bars represent standard deviations. Statistical analysis was performed by one-way ANOVA, with multiple comparisons between each pH value with BHI: ****, P<0.0001. If not indicated, differences are not significant.


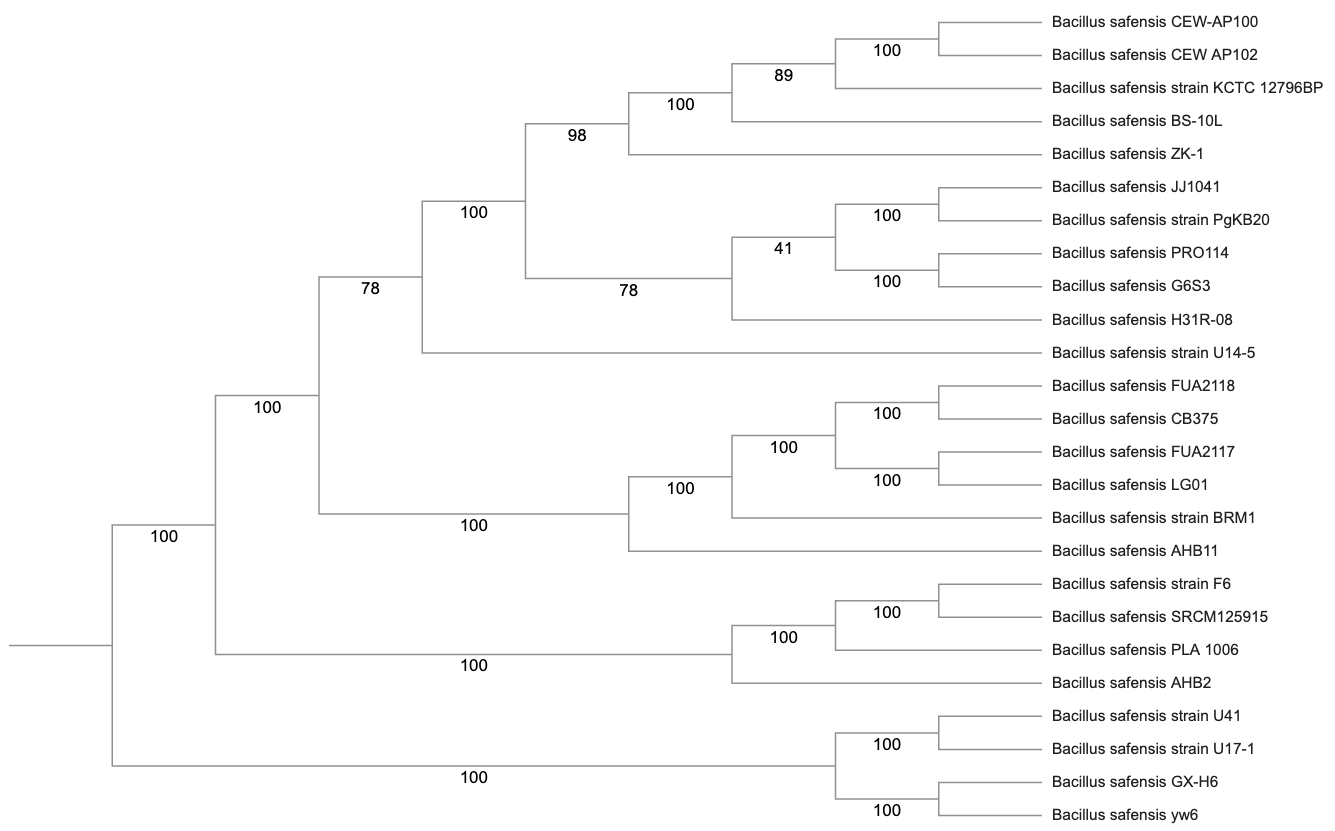


**Figure S4:** Phylogenetic tree of *B. safensis* CB375 and closely related strains. *B. safensis* CB375 genome data was used as a query to find similar genomes in the BV-BRC database. The top twenty-five *B. safensis* hits with complete genome assembly were chosen to construct a phylogenetic tree with strain CB375, using BV-BRC Phylogenetic Tree Building service.

CB375 MFKQ-IDENYPRKEHFHHYMKVTRCSYSLVIDLDITKLHSILKEKKLKVYPVQIYLLARA 59

CAT-S NFNKIDLDNWKRKEIFNHYLNQ-QTTFSITTEIDISVLYRNIKQEGYKFYPAFIFLVTRV 59

*:: :*: *** *:**:: : ::*:. ::**: *: :*:: *.**. *:*::*.

CB375 VQKIPEFRMDLV-NDELGHWDLLHPSYTILNKAAKTFSSIWTPYDENFARFYKSCVADIE 118

CAT-S INSNTAFRTGYNSDGELGYWDKLEPLYTIFDGVSKTFSGIWTPVKNDFKEFYDLYLSDVE 119

::. ** . :.***:** *.* ***:: .:****.**** .::* .**. ::*:*

CB375 TYSESNKLFPKPNMPENMFNISSLPWIDFTSFNLNVSTDETYLLPIFTLGQFKMKGEKII 178

CAT-S KYNGSGKLFPKTPIPENAFSLSIIPWTSFTGFNLNINNNSNYLLPIITAGKFINKGNSIY 179

.*. *.***** :*** *.:* :** .**.****:..:..*****:* *:* **:.*

CB375 LPVAIQVHHAVCDGYHVGQYVEYLRWLIEHCEEWLSDSLYT 219

CAT-S LPLSLQVHHSVCDGYHAGLFMNSIQELSDRPNDWLL----- 215

**:::****:******.* ::: :: * :: ::**

**Figure S5:** Sequence alignment of chloramphenicol acetyltransferase (CAT) detected in *B. safensis* CB375 (denoted as CB375), with chloramphenicol acetyltransferase from commonly used expression plasmids (denoted as CAT-S, GenBank ID: ACO90425). * indicates identical amino acid residues, : indicates similar residues.


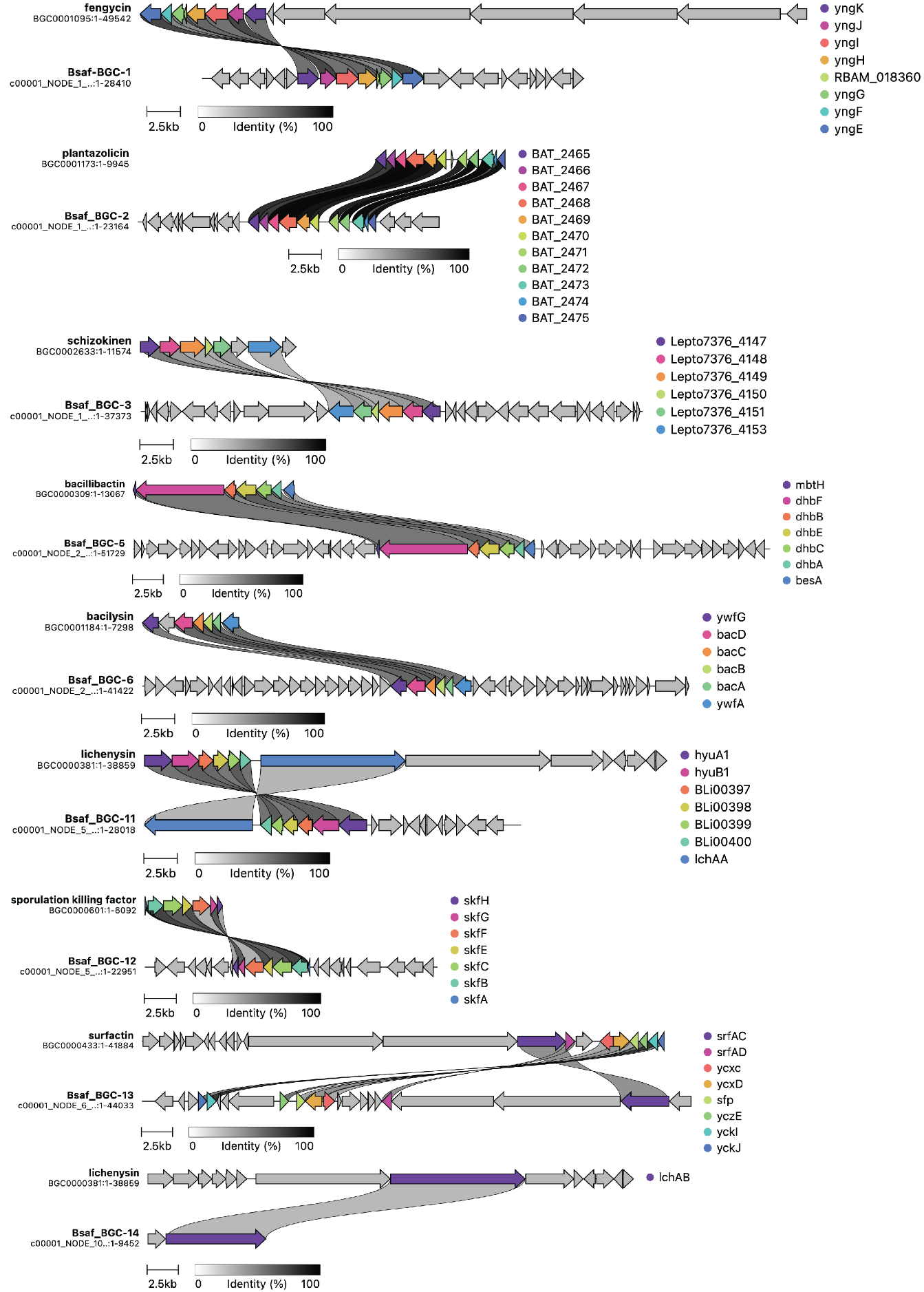


**Figure S6:** Alignments of Biosynthetic Gene Clusters in *B. safensis* CB375 with known clusters

**Figure S7:** Representative growth curves of *L. monocytogenes* cultures, grown in BHI broth at 37°C with aeration, in with cell-free supernatants from *B. safensis* or *L. monocytogenes*.


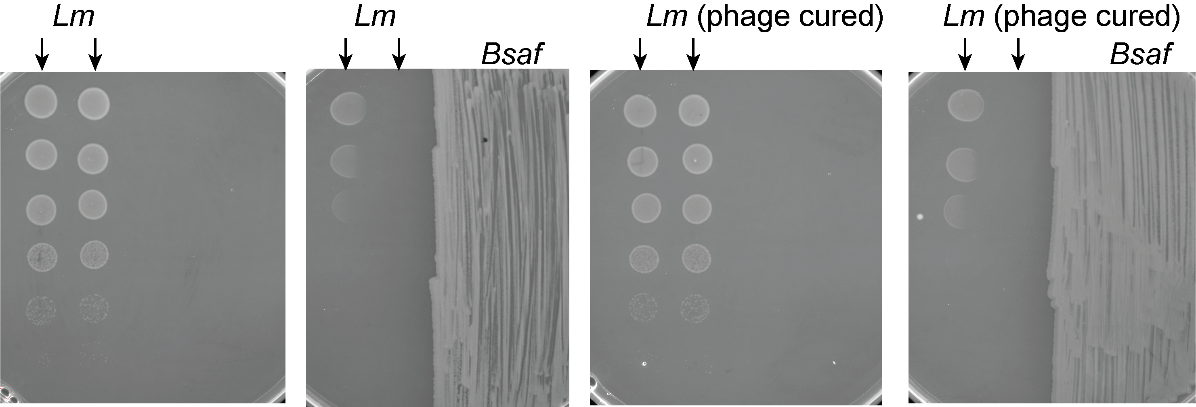


**Figure S8:** Inhibition of *L. monocytogenes* by *B. safensis*. *L. monocytogenes* strain 10403S and an isogenic strain that is cured of the Φ10403S prophage. *B. safensis* was incubated for 2 days prior to spotting of *L. monocytogenes* in the other half of the agar.

**Table S1:** Average nucleotide identity analysis of *B. safensis* CB375 with other *B. safensis* strains that have complete genome assembly

| ***Bacillus safensis* strain** | **Average nucleotide identity compared to CB375** |
| --- | --- |
| FUA2118 | 98.8749 |
| BRM1 | 98.8377 |
| FUA2117 | 98.7097 |
| LG01 | 98.6875 |
| AHB11 | 98.4698 |
| U14-5 | 97.36 |
| JJ1041 | 97.3426 |
| PRO114 | 97.3241 |
| PgKB20 | 97.3123 |
| G6S3 | 97.3013 |
| ZK-1 | 97.2911 |
| CEW-AP100 | 97.2682 |
| CEW_AP102 | 97.2606 |
| KCTC_12796BP | 97.2554 |
| H31R-08 | 97.2494 |
| BS-10L | 97.2085 |
| SRCM125915 | 96.3902 |
| F6 | 96.3047 |
| PLA_1006 | 96.2962 |
| AHB2 | 96.2813 |
| yw6 | 96.0488 |
| U41 | 95.9989 |
| U17-1 | 95.9967 |
| GX-H6 | 95.9887 |
